# Influence of preanalytical variables on performance of delta-like protein 3 (DLL3) predictive immunohistochemistry

**DOI:** 10.1007/s00428-020-02848-y

**Published:** 2020-06-02

**Authors:** Teodora Radonic, S. Duin, W. Vos, P. Kortman, Aeilko H. Zwinderman, Erik Thunnissen

**Affiliations:** 1 Department of Pathology, Amsterdam University Medical Center, De Boelelaan, 1117 Amsterdam, The Netherlands; 2 Department of Clinical Epidemiology and Biostatistics, Amsterdam University Medical Centers, Meibergdreef 9, 1105 AZ Amsterdam, The Netherlands

**Keywords:** DLL3, Immunohistochemistry, Preanalytical variable, Formalin, Decalcification

## Abstract

DLL3 might become a predictive immunohistochemical marker in small cell carcinoma of the lung (SCLC). We investigated the influence of pre-analytical handling of samples on the performance of DLL3 immunohistochemistry (IHC) using DLL3 SP347 ready to use assay (Ventana). DLL3 positive cell lines were subjected to different experimental conditions mimicking the pre-analytical variation in daily clinical practice. Formalin fixation of 24 h led to the most optimal results of DLL3 IHC. Longstanding fixation in Cytolyt, methanol-based fixative for cytology samples, but also decalcification using a mix of formic- and hydrochloracid resulted in decreased DLL3 staining. Postponed staining of blanc slides for 3 months also decreased DLL3 IHC. Postponed fixation of the SCLC cell lines did not influence the performance of DLL3 IHC, although this might be different in the tissues than in the cell lines. In conclusion, different pre-analytical variables decrease the performance of DLL3 IHC. These findings are essential for implementing novel predictive immunohistochemical biomarkers in daily pathology practice.

**Electronic supplementary material:**

The online version of this article (10.1007/s00428-020-02848-y) contains supplementary material, which is available to authorized users.

## Introduction

DLL3 emerged as a potential target in small cell lung cancer (SCLC) from gene expression studies. DLL3 is a member of a Notch ligand receptor family and in normal cells inhibits the Notch pathway [1, 2] which is frequently inactivated in SCLC [3]. DLL3 is overexpressed in most SCLC cases on cell membrane and intracytoplasmatically about 35-fold compared to normal lung in the majority of patients with SCLC [4]. Moreover, DLL3 is not expressed in the normal tissues in adults and is therefore an interesting therapeutic target in SCLC [4].

From the experience gained with PD-L1 as a widely used predictive IHC biomarker in pathology, it was recognized that preanalytical, analytical, and postanalytical variables can influence testing performance [5–8]. Pre-analytical variables in daily pathology practice include steps from the sample collection until start of fixation. Potentially, most important factors for the quality of the sample and those that can be influenced by the way of sample handling are (1) warm ischemia time (relevant for the resection specimens and starts from the blood supply cut off), (2) cold ischemia time (time from removal from the body until fixation) [8], (3) temperature at which fresh sample is kept during cold ischemia time [9], (4) type of fixative and decalcification [10], but also (5) tissue to fixative ratio [11]. In general, the possible effect of variation in prenanalytical variables on predictive biomarker testing is frequently mentioned in reviews [12, 13], but detailed studies on this topic are sparse. Recently, in a tissue model experiment on the effect of delayed fixation, reduced IHC staining of several antibodies, including PD-L1 and cytokeratins, was found [8]. Moreover, in another study, the type and duration of decalcification were shown to drastically reduce the PD-L1 IHC intensity and the proportion of positive cells [10, 14]. When PD-L1 IHC intensities after decalcification were compared between two IHC clones (22C3 and E1L3N), there was a slight reduction in percentage of positive cells only for 22C3 clone while staining using E1L3N clone was stable [10]. Therefore, the knowledge of the effect of preanalytical variables on the performance of a predictive biomarker is essential for implementation in the clinical practice.

The aim of this study is to examine the influence of different clinically relevant preanalytical variables on the performance of DLL3 IHC. We assessed the effect of different fixatives, fixation times, delay in fixation, and use of a decalcification procedure.

## Methods

### Cell lines and culture

Three cell lines were used: NCI-H69 [H69] (ATCC® HTB119™) human SCLC line reported to have a low DLL3 expression; NCI-H82 [H82] (ATCC® HTB175™) human SCLC line reported to have a medium DLL3 expression, comparable with that in patient SCLC samples and A549 (ATCC® CCL-185™); and human non-small cell cancer line previously reported to have virtually no DLL3 expression (negative control) [15].

Three cell lines were separately grown under aseptic conditions in an incubator providing a humidified atmosphere of 5% CO2 in air. All cell lines were used between 3 and 9 passages after thawing to ensure complete revival and routinely tested for mycoplasma. All cell lines were cultured in RPMI base media supplemented with 10% fetal bovine serum, 2 mM L-glutamine, 10 mM HEPES, 1 mM sodium pyruvate, 4500 mg/L glucose, 1500 mg/L sodium bicarbonate, and 100 U/mL penicillin and 100 μg/mL streptomycin.

After sufficient cells were cultured, H69 and H82 were centrifuged and subsequently objected to different conditions as described below. A549 was first treated with trypsin, and two times washed in PBS. All three cell lines were embedded in agar gel, processed in TissueTek Xpress X120 (Sakura Finetek USA, Torrance, CA), and embedded in paraffin block.

### Preanalytical conditions

In Table 1 all preanalytical conditions are presented. In the first phase, the optimal fixation time for two fixatives was determined. The fixatives were 10% neutral buffered formalin for tissues and Cytolyt®ThinPrep (Hologic Inc., Marlborough, MA, USA), a methanol-based medium for cytology specimens. After this step, the fixation medium and time were selected that resulted in highest DLL3 IHC staining as “golden standard” for the next steps. In order to examine whether formalin postfixation could modify DLL3 intensity after initial Cytolyt fixation, we tested two different times of Cytolyt fixation followed by two different times of formalin postfixation.

**Table 1:** Preanalytical conditions

Preanalytical variable		Times (h)*		
Fixation in formalin		3, 24, 48, 72		
Fixation in Cytolyt		3, 24, 48, 72		
Fixation in Cytolyt followed by formalin				
	Time in Cytolyt	3	24	24
	Subsequent time in formalin	24	3	24
Postponed fixation				
	Room temperature	3, 6, 24		
	4 °C	3, 6, 24		
Postponed staining				
	Room temperature	1 week, 3 months		
	4 °C	1 week, 3 months		
Decalcification Kris.		24, 48		
Decalcification Sakura		24, 48		

*Unless otherwise specified

*Kris*. Kristensen

In the following phase, postponed fixation was examined. The cells were centrifuged, washed, and left without bovine serum and without fixative for 3, 6, and 24 h on room temperature and 4 °C, respectively. Subsequently, cells were processed using the golden standard method which emerged from the previous step (24-h formalin fixation on room temperature).

Influence of decalcification was tested with two commonly used decalcification media:(1) 1:1 8 N-formic acid and 1-N sodium formate solution (pH 2.2), also known as Kristensen and (2) Sakura Reagent TDE30 decalcifier. In line with the clinical practice specimens were first fixated and subsequently decalcified. Different fixation times (3, 24, or 48 h) in 10% buffered formalin were examined with subsequent longstanding decalcification for 24 or 48 h.

Influence of postponed staining of blanc histological sections was examined, where the blancs were left on the room temperature or 4 °C for 1 week and 3 months, respectively.

The whole series of experiments (including growing the cell cultures) was performed in duplo.

### Immunohistochemistry

From each block, 4-μm thick sections were cut and mounted on positively charged glass slides and stained using DLL3 ready-to-use assay (clone SP347, Ventana, Roche, Tucson, AZ, USA), after pre-treatment with CC1 for 80 min, and using the OptiView detection kit (Ventana) on a Benchmark Ultra slide staining instrument (Ventana), following the manufacturer’s instructions.

Staining intensity was scored using the H-score (range 0–300). In short, the percentage of cells at each staining intensity level (0, 1+, 2+, and 3+) is scored, and H-score is calculated using the following formula: 1 × (% cells 1+) + 2 × (% cells 2+) + 3 × (% cells 3+) [16].

All slides were stained twice (in duplo staining) and scored by two investigators (in duplo reading).

### Statistics

H-scores were summarized using means, medians, and standard deviations (SD). Statistical analysis was performed using R [17]. Mixed-effects linear regression was used to examine the differences between the experimental conditions. The duplo experiments and the two observers were treated as two-by-two clustered observations within the experimental conditions. A *p* value of < 0.05 was considered to be statistically significant.

## Results

**Fig. 1:**
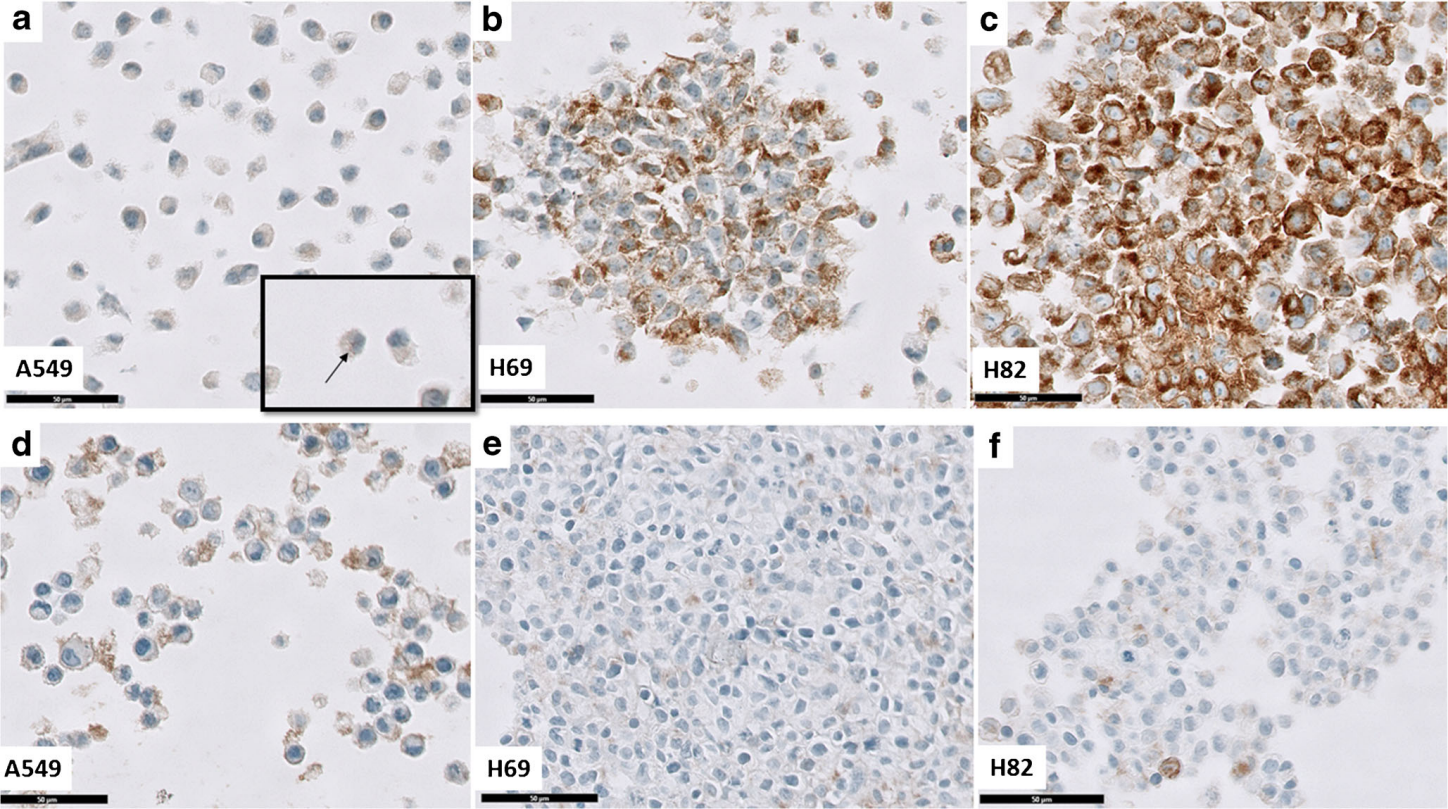
**a**–**c** DLL3 IHC staining (× 40 magnification, black bar down left 50 μm) in the cell lines using the optimal fixation method (24 h in 10% buffered formalin). A549 was negative for DLL3 with few dots occasionally noted around the nucleus (arrow). Note the fine granular cytoplasmic positivity at intermediate (H69) and high (H82) intensity level. **d**–**f** DLL3 staining in the cell lines after decalcification in Sakura. Note the artefactual membranous positivity in A549 (**d**). Also note a significant reduction in DLL3 staining in H69 and H82 when compared to the optimal fixation method (**b**, **c**)

DLL3 IHC was positive in the cell lines at the expected intensity: A549 was negative with occasionally a few dots around the nucleus at the highest magnification (× 40) (Fig. 1a), most probably a physiological staining in endoplasmic reticulum [2, 17]. DLL3 staining in H69 and H82 cell lines was granular cytoplasmatic with higher DLL3 intensity in H82 and low in H69 (Fig. 1b, c). We found a significant difference in DLL3 staining between in duplo experiments (mean H score of experiment 1 was 94, SD = 103, mean H score and SD of experiment 2 was 88, SD = 102 *p* = 0.002) and the scores of two investigators (mean H score investigator 1 was 97 SD = 105, mean H score investigator 2 was 86 SD = 101, *p* = 0.0001). Since these differences were systematic and comparable in all experimental conditions (Supplementary Fig. 1 and 2), we averaged the H-scores per condition.

The differences between the 25 tested experimental conditions (as listed in Table 1) were statistically significant (*p* < 0.0001) with the interclass correlations between the H-scores within the conditions of 0.59 for H69 and 0.77 for H82.

### Variation in fixatives and fixation times

**Fig. 2:**
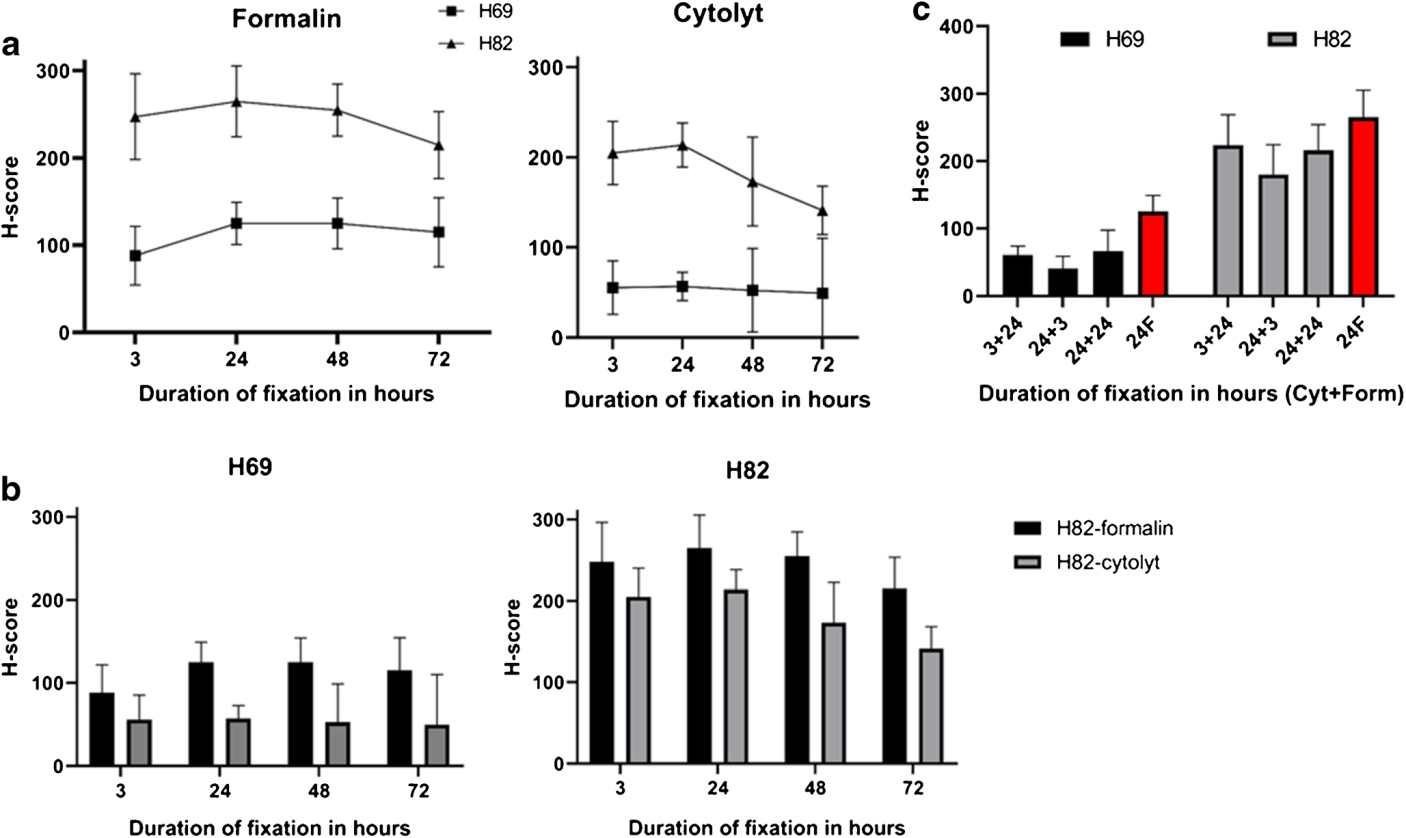
**a**, **b** DLL3 IHC intensity (mean and SD of H-score, *x* axis) after different fixation times (in hours, *y* axis) in formalin (black bars) and Cytolyt (gray bars) plotted for both cell lines. Note the lower DLL3 intensity after fixation in Cytolyt whereas the difference became larger in the medium/low DLL3 intensity cell line (H69) and increases with fixation time in Cytolyt. Note also the maximal intensity after 24 h of formalin fixation, considered as a golden standard for further experiments. **c** DLL3 IHC intensities (H-score, *x* axis) plotted per positive cell line after initial fixation in Cytolyt and postfixation in formalin for different times (*y* axis). Per time point Cytolyt and formalin times are depicted. In red column, the intensity of the same cell line after 24 h of formalin fixation (golden standard). Note how DLL3 intensity could be partially restored by postfixation in formalin of 24 h

All three cell lines were subjected to fixation in 10% neutral buffered formalin and CytoLyt (Fig. 2a) for four different times (3, 24, 48, and 72 h). Formalin fixation of 24 h showed the highest H-scores and was considered the golden standard for further experiments. Fixation time of 3 h in formalin was already sufficient, and DLL3 staining was comparable after longstanding fixation (48 and 72 h) (*p* = 0.33 for H82; *p* = 0.28 for H69). Overall, DLL3 staining was lower after Cytolyt fixation compared with formalin in both cell lines in all conditions with the difference becoming more prominent after Cytolyt fixation of longer than 24 h (*p* = 0.0021 for H82; *p* < 0.001 for H69).

### Influence of formalin postfixation after CytoLyt

The influence of postfixation in formalin was examined after the initial fixation in CytoLyt for three different times: 3 h CytoLyt followed by 24-h formalin (3C + 24F, see Table 1); 24-h CytoLyt followed by 3-h formalin (24C + 3F); and 24 h (24C + 24F, see Table 1) of formalin postfixation (Fig. 2b). We showed that postfixation in formalin for 24 h restored to a certain extent the IHC intensity irrespective of the duration of Cytolyt prefixation (three or 24 h: *p* = 0.0349 for H69; *p* = 0.59 for H82) but not to the level of the golden standard (24 h formalin fixation), although the differences were not statistically significant but showed a certain trend (*p* = 0.063). Interestingly, we noted a larger difference in the low-DLL3 expression cell line H69.

### Postponed fixation

Cells were centrifuged, washed, and left without bovine serum and without fixative for 3, 6, and 24 h on room temperature and 4 °C followed by the golden standard fixation time of 24 h formalin (Suppl Fig. 3). There were no differences observed, irrespective of the duration of fixation postponement (*p* = 0.23 for H69; *p* = 0.70 for H82) or the temperature (*p* = 0.55 for H69; *p* = 0.74 for H82).

### Decalcification

**Fig. 3:**
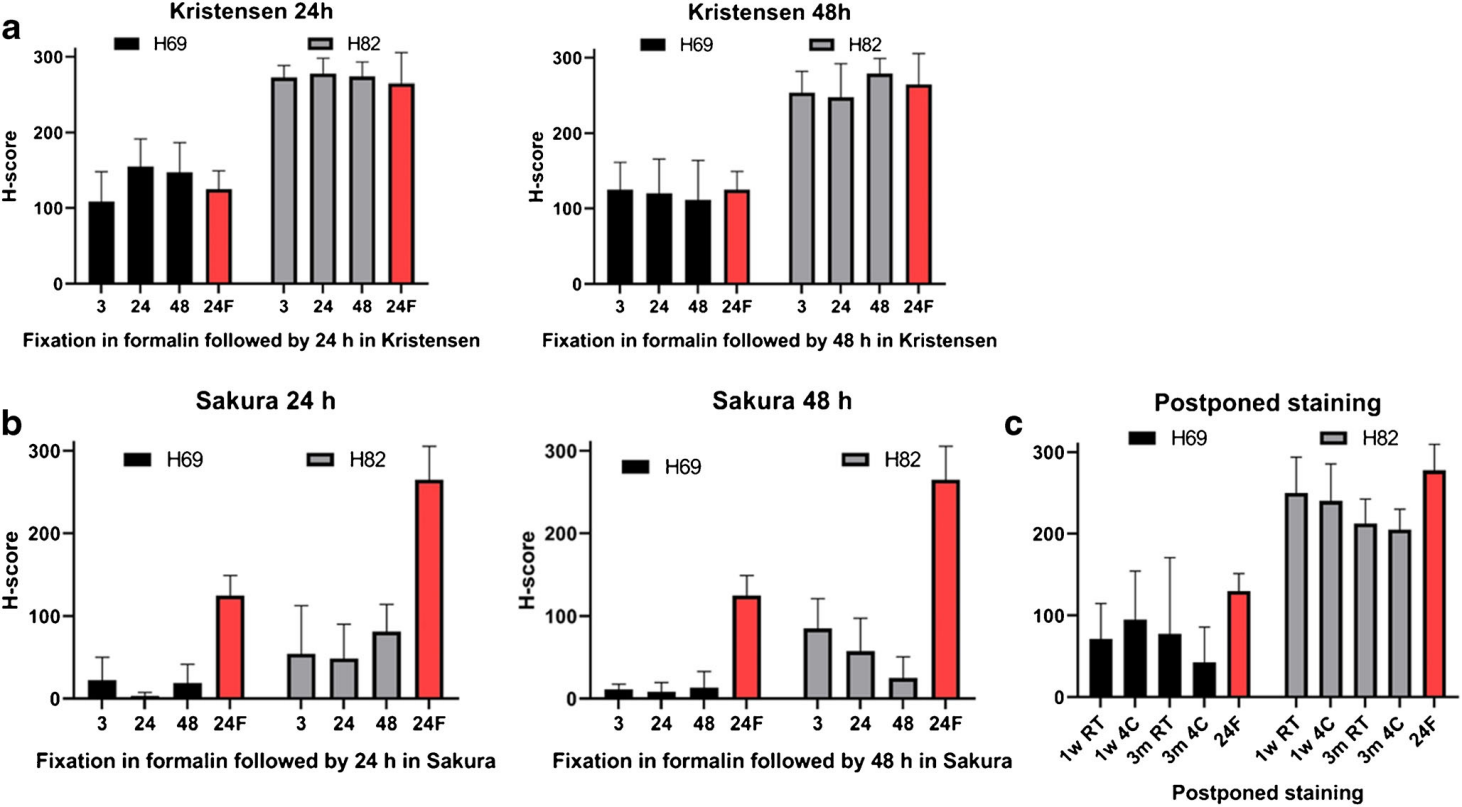
DLL3 staining intensity (mean and SD of H-score, *x* axis) after decalcification for 24 and 48 h tested on high expressing (H82) and low (H69) DLL3 expressing cell lines with **a** Kristensen and **b** Sakura decalcification procedure. Cells underwent different formalin fixation times, shown in hours on the *x* axis. **c** DLL3 staining after postponed staining of blanc slides for 1 week or 3 months at room temperature (RT) or 4C (*y* axis). In every panel DLL3 staining after optimal fixation is shown in red (24F)

Different times of formalin fixation were followed by two times of decalcification (24 and 48 h) using the two decalcification methods (Kristensen and Sakura). We showed that decalcification in Kristensen did not lead to decrease in DLL3 staining intensity (Fig. 3a) (*p* = 0.50). In contrast, decalcification in Sakura led to significant decrease in DLL3 intensity in all tested times (Fig. 3b) (*p* < 0.0001 and *p* < 0.0001 at 24 h for H69; *p* = 0.0004 at 24 h and *p* = 0.0006 at 48 h for H82). Moreover, we observed an artefactual positivity in the DLL3 negative cell line (A549) after Sakura decalcification of any duration (Fig. 1b). The staining was membranous but not cytoplasmatic granular as in the DLL3 positive cell lines under normal conditions. In the same figure, we demonstrate the influence of Sakura decalcification on DLL3 intensity of DLL3 positive cell lines.

### Postponed staining

Postponement of IHC of blanc slides left unstained of either 1 week or 3 months led to a decrease of the DLL3 staining intensity which was statistically non-significant (*p* = 0.51 for H69; *p* = 0.31 for H82) (Fig. 3c). The difference was slightly larger after 3 months of staining postponement. Interestingly, temperature at which blanc slides were stored (room temperature or 4 °C), resulted in a similar (statistically non-significant: *p* = 0.81 for H69, *p* = 0.49 for H82) decrease in staining intensity.

## Discussion

The influence of preanalytical variables on the staining intensity of DLL3 IHC revealed that formalin fixation of 24 h led to an optimal performance of DLL3 IHC staining and that longstanding fixation with Cytolyt as well as decalcification using Sakura Reagent TDE30 decalcifier resulted in essential reduction of DLL3 staining intensities.

From what is reported in the literature, the diagnosis of SCLC is made on cytology samples in 15–31% patients as many patients have a broadly metastasized disease at the time of diagnosis [18]. These percentages may vary even more in the clinical practice then reported in the literature. Fixation in Cytolyt, preservative solution used in our institution, resulted in overall lower DLL3 IHC intensities than formalin-fixed cell lines. The contents of the fine needle aspiration are typically expelled and rinsed in a cell preservative solution, in order to assure sufficient material for acquisition of a cell block for IHC [19]. In daily practice, a fine needle aspirate is preserved in Cytolyt, a methanol-based fixative, until a cell block is made for immunocytochemistry. During this last step, the samples are commonly subjected to formalin postfixation before being embedded in paraffin [20]. In an experiment mimicking this situation, reduced DLL3 intensity after Cytolyt fixation alone, could partially be restored by postfixation for 24 h in neutral buffered formalin. In a comparable study on PD-L1 IHC after different fixation methods, Cytolyt-fixed cytology samples and histology samples from the same tumor had comparable percentage of positive tumor cells in PD-L1 IHC when the tissue block is made using agar-based technique where formalin fixation of the embedded cells is one of the steps [21]. In contrast, when only methanol-based fixative was used (Celient technique), cytology samples were proportionally more often PD-L1 negative compared to their histological control [21]. Our experiment and these data support the finding that formalin (post)fixation is essential for the optimal performance of DLL3 IHC.

Antigen retrieval is an important step in the process of immunohistochemistry and is mainly based on heating induced protein modifications in order to release the antigen from methylene bridges formed between the proteins by formalin [22]. Some antigen retrieval procedures include pH modifications during heating in order to enhance the effect [23, 24]. In case of alcohol-based fixatives, proteins undergo coagulation instead of forming of methylene bridges, and antigen retrieval developed for formalin-fixed tissues is ineffective resulting in lower IHC intensities [25]. Our findings indicate that predictive immunohistochemistry should be validated on cytology samples and that formalin fixation of cytology samples leads to improvement. It remains to be investigated whether these lower DLL3 intensities in cytology fixative are clinically relevant. After showing promising results in phase 1 and 2 trials [26–28], Rova-T, a monoclonal antiDLL3 antibody-drug conjugate, failed to prove therapeutic benefit in phase 3 trials [27, 28]. Currently, DLL3 is being investigated in a phase 1 trial using bispecific T cell engager (BiTE) antibody construct targeting DLL3 [29], a T cell redirecting immunotherapy. BiTE is designed to transiently connect DLL3-positive cells to CD3-positive T cells and induce T cell-mediated cell lysis and concomitant T cell proliferation.

We also demonstrated that type of decalcification can drastically decrease the performance of the IHC. Sakura is currently used in small biopsies for short decalcification (1–2 h) and contains a mix of formic acid and hydrochloric acid with pH of 0.1 [30]. The Kristensen method is based on a mix of formic acid and its sodium salt, sodium formate, and has a higher pH = 2.2, and is used for larger bone fragments which need longstanding decalcification [31]. This difference in acidity may be a reason for the better performance of Kristensen-decalcified samples. It should be noted that in our experiments, a longer Sakura decalcification time was applied than recommended by the Sakura manufacturer, and should be avoided in the daily practice. Artefactual positivity of the DLL3 negative cell line after Sakura decalcification is an important finding for the clinical practice. Our results indicate that IHC, especially the predictive IHC, should be validated using different methods of decalcification. It should be noted though that our study did not include all the decalcifying methods, as for example, the ethylenediaminetetracetic acid (EDTA) which is commonly used for small biopsies by pathology laboratories and has a higher pH.

In our previous work, we demonstrated that postponed fixation of lung resection specimens resulted in a significant reduction of different IHC as cytokeratins, TTF1, but most importantly of PD-L1 [8]. The proportion of PD-L1-positive samples reduced dramatically with the duration of cold ischemia. In the clinical practice, predictive IHC variability can lead to patients not be considered eligible for immune therapy and checkpoint inhibitors [32]. Interestingly, DLL3 was not sensitive for postponed fixation (cold ischemia). Possibly, the DLL3 protein is relatively stable over time, although the protein half-life is not known. Another explanation could be that the cell lines are less sensitive to cold ischemia due to the lack of the tissue context with inflammatory cells and stroma which produce and release degrading enzymes.

Postponed staining reduced the performance of the DLL3 staining irrespective of the temperature at which the blanc slides were kept. After the slides of 4 μm are cut and put on the glass, tissue is exposed to oxygen from the air. We hypothesize that oxidation of the proteins might lead to reduced staining, as described earlier [33]. Although this experiment was not statistically significant, it might prove clinically relevant depending on the thresholds of DLL3 positivity in the clinical trials. Interestingly, in another study, when postponed IHC was investigated on 32 IHC markers (postponement of blanc slides staining up to 1 year), four of 32 IHC markers showed reduced staining when the blanc slides were kept at 4C compared with 11 and 21 markers with reduced staining when the slides were kept on respectively room temperature and 37C, indicating that epitope stability of some IHC markers is dependent on the temperature [34].

Notably, most differences between the conditions were observed in the H69 cell line, which has a low DLL3 epitope concentration. Samples with low epitope concentration (critical samples) are considered optimal for validation of novel IHC tests for an equivalent outcome compared to the clinically validated test, because a slight variation in the test can lead to the altered outcome [32, 35]. This is in contrast to the samples with high epitope concentration, which will reach the maximum staining irrespective of the slight variations in the test used. Similarly, H69 cell line could be considered a “critical sample” for preanalytical conditions testing and their influence on DLL3 IHC. Differences between the conditions were more easily detected in a cell line with a low DLL3 epitope concentration.

In conclusion, formalin fixation of 24 h led to the most optimal results of DLL3 IHC. Acceptable staining was reached already after 3 h of formalin fixation. Fixation in Cytolyt, longstanding decalcification using Sakura, and postponed staining of blanc slides led to decreased DLL3 IHC. It remains to be determined whether these differences will be clinically relevant when DLL3 is implemented in the clinical practice.

## Electronic supplementary material

Suppl Fig 1 H-score variation between the experiments, plotted per cell line. Note the systematic differences between the two experiments. (PDF 5 kb)

Suppl Figure 2 Differences between the two raters (in duplo reading) were also systematic with a slight bigger differences in H69 (low DLL3 epitope concentration cell line). A, B. Plots of the mean H-scores of the two raters (each axis one rater). C,D. differences in H-score between two raters plotted against the mean H-scores between the raters. Note the systematic differences between the raters. (PDF 7 kb)

Supplementary Figure 3 When fixation was postponed for 3,6,24 hours (x-axis), there was no decrease of DLL3 staining found, irrespective of the temperature at which the cells were kept during this cold ischemia time. Red bars represent the DLL3 levels of both cell lines when golden standard of fixation is used (24 hours formalin). (PNG 120 kb)

High resolution image (TIF 16905 kb)
