# Supplementary figures and images for: Influence of preanalytical variables on performance of delta-like protein 3 (DLL3) predictive immunohistochemistry
Source: Virchows Arch. 2020 Jun 2;478(2):293–300. doi: 10.1007/s00428-020-02848-y (PMC7969697; doi:10.1007/s00428-020-02848-y)

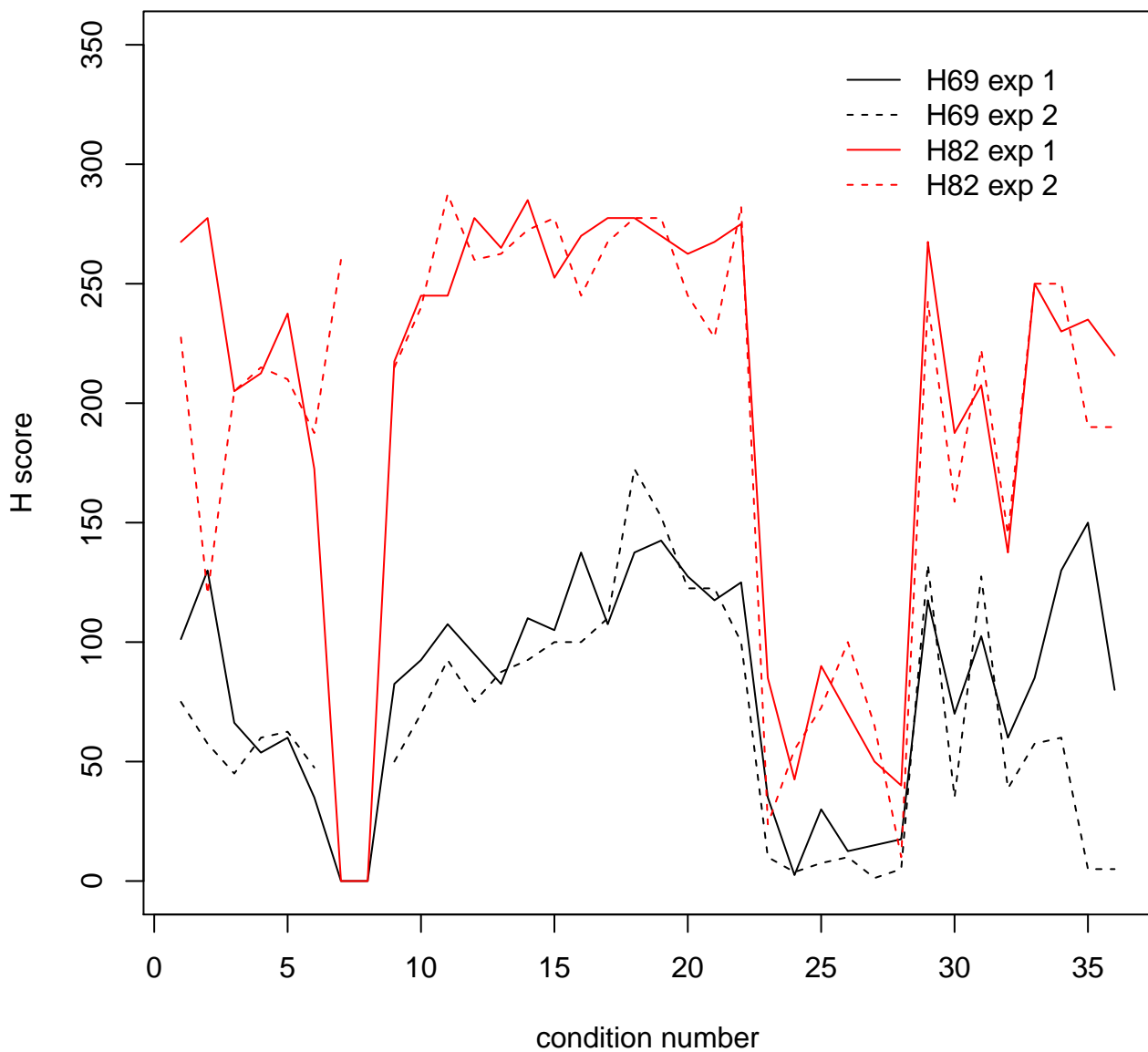

Supplement: Supplementary file 1 — H-score variation between the experiments, plotted per cell line. Note the systematic differences between the two experiments. (PDF 5 kb) [file 428_2020_2848_MOESM1_ESM.pdf]

**H69**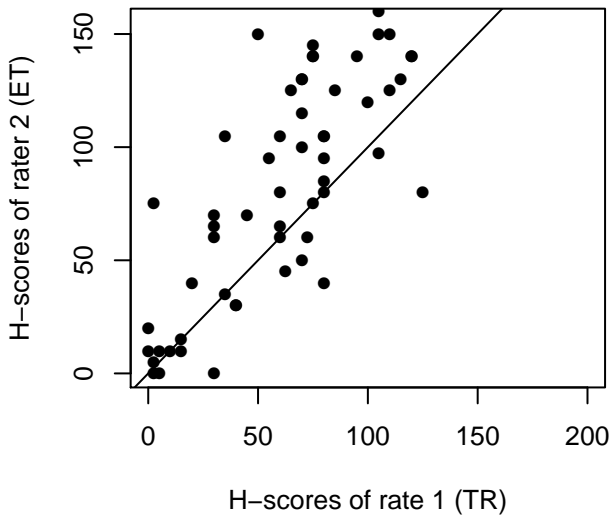**H69**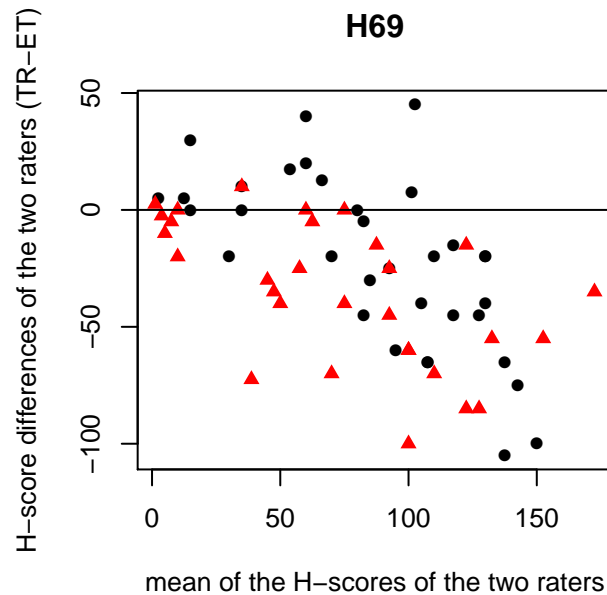**H82**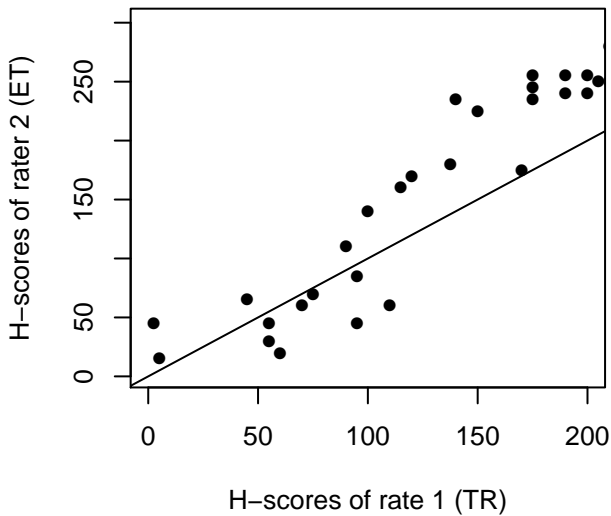**H82**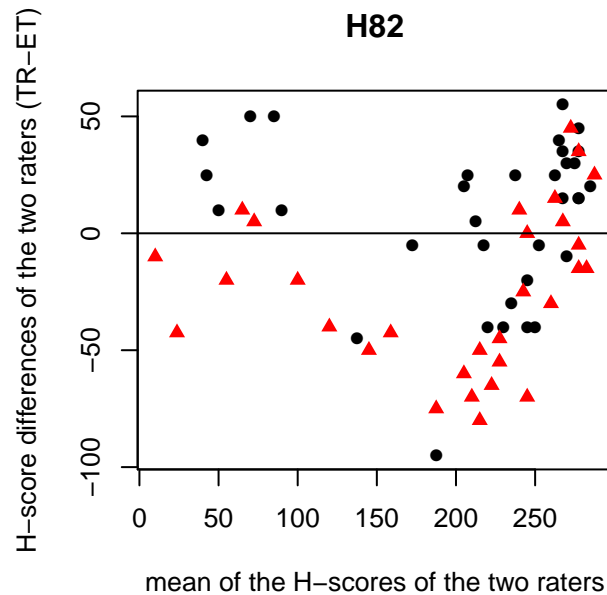

Supplement: Supplementary file 2 — Differences between the two raters (in duplo reading) were also systematic with a slight bigger differences in H69 (low DLL3 epitope concentration cell line). A, B. Plots of the mean H-scores of the two raters (each axis one rater). C,D. differences in H-score between two raters plotted against the mean H-scores between the raters. Note the systematic differences between the raters. (PDF 7 kb) [file 428_2020_2848_MOESM2_ESM.pdf]

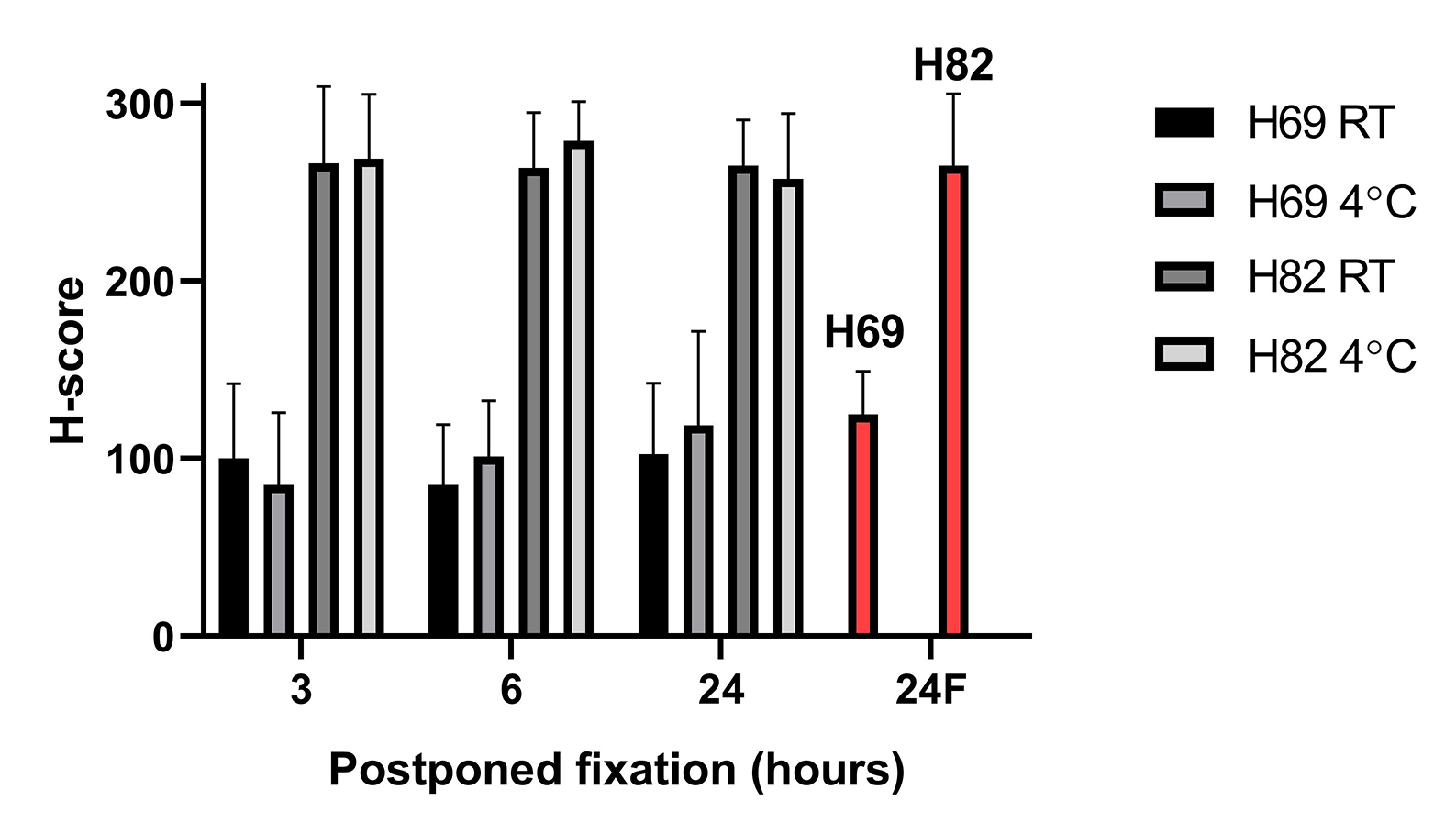

Supplement: Supplementary file 3 — When fixation was postponed for 3,6,24 hours (x-axis), there was no decrease of DLL3 staining found, irrespective of the temperature at which the cells were kept during this cold ischemia time. Red bars represent the DLL3 levels of both cell lines when golden standard of fixation is used (24 hours formalin). (PNG 120 kb) [file 428_2020_2848_Fig4_ESM.png]

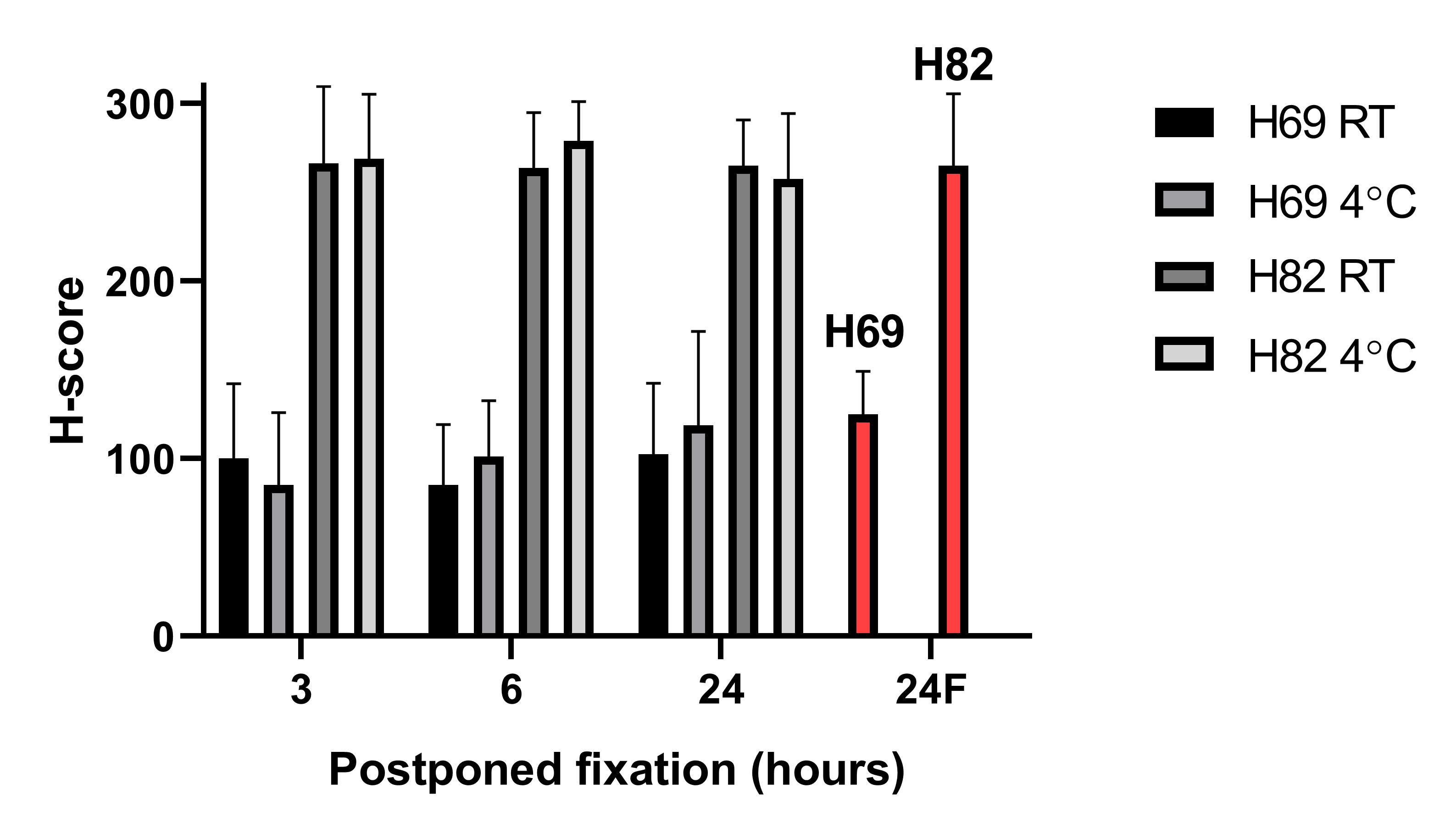

Supplement: Supplementary file 4 — High resolution image (TIF 16905 kb) [file 428_2020_2848_MOESM3_ESM.tif]
